# Electrostatic treatment enhances pollen viability and improves fruit quality in ‘Xuxiang’ kiwifruit

**DOI:** 10.3389/fpls.2026.1769809

**Published:** 2026-03-30

**Authors:** Junyu Sun, Zhihao Zhang, Yanjun Fang, Yufei Zhang, Yimeng Li, Yaming Wu, Yanfei Liu, Zhande Liu, Fuxi Shi

**Affiliations:** 1 College of Mechanical and Electronic Engineering, Northwest Agriculture and Forestry (A&F) University, Yangling, Shaanxi, China; 2 State Key Laboratory for Crop Stress Resistance and High-Efficiency Production, College of Life Sciences, Northwest Agriculture and Forestry (A&F) University, Yangling, Shaanxi, China; 3 State Key Laboratory of Plant Cell and Chromosome Engineering, Institute of Genetics and Developmental Biology, Chinese Academy of Sciences, Beijing, China; 4 College of Food Science and Engineering, Northwest Agriculture and Forestry (A&F) University, Yangling, Shaanxi, China; 5 College of Horticulture, Northwest Agriculture and Forestry (A&F) University, Yangling, Shaanxi, China

**Keywords:** abiotic stress, fruit quality assessment, kiwifruit pollination, orchard cultivation technique, pollination technology

## Abstract

Electrostatic treatment holds promise for enhancing pollen and seed viability, as well as improving crop quality. However, there are limited reports regarding the effects of electrostatically treated pollen on pollination and fruit quality in plants. In this study, an electrostatic pollination platform was established, treated pollen was used to pollinate the kiwifruit cultivar (*Actinidia deliciosa* cv. ‘Xuxiang’), and in vitro germination experiments were conducted. In field experiment, style samples were collected every 0.5 hours over a continuous period of 20 hours, and pollen tube behavior was observed using fluorescence staining. Sufficient kiwifruit samples were harvested to determine the contents of sugars, acids, and volatile organic compounds (VOCs) in fruits with varying firmness. Electrostatic treatment increased pollen germination rate, accelerated pollen tube growth to style base by 5 hours. During both harvest and ripening stages, soluble solids content (SSC) and sugar content (fructose, glucose, and sucrose) significantly increased (up to 5.6%), while dry matter content (DM) showed a slight increase, titratable acidity (TA) and vitamin C (VC) content decreased, therefore, kiwifruit under electrostatic pollination exhibits a sweeter flavor profile, suggesting 28 kV to harvest superior-quality kiwifruit. Interestingly, in ripening phase, varying electrostatic voltages influence the content of VOCs, particularly esters, suggesting that different voltages may impart distinct aroma profiles to kiwifruit. This study proposes a novel orchard management and cultivation technique.

## 1 Introduction

Kiwifruit (*Actinidia chinensis Planch*.) belongs to the genus Actinidia of the family Actinidiaceae. As dioecious fruit trees, effective pollination and fertilization are essential for fruit set and form the basis for subsequent high quality and yield. Under natural conditions, pollination in kiwifruit occurs via anemophily (wind pollination) and entomophily (insect pollination) (Vaissiére et al., 1996; Testolin et al., 1991). However, anemophilous pollination is often constrained by biological and physical factors, for instance, the relatively high mass of pollen grains (González et al., 1995; Labouche et al., 2017) limits its effective dispersal distance (Meléndez-Ramírez et al., 2004; Hall and Walter, 2018). Entomophilous pollination faces additional challenges due to asynchrony in flowering times between male and female kiwifruit cultivars (Razeto et al., 2005), as well as the high susceptibility of insect pollinators to pesticides (Brown et al., 2018; Overturf et al., 2022). Currently, global warming has given rise to anomalous climatic conditions during flowering periods (Gurbuz et al., 2024; Patterson et al., 1999). Previous study indicates that climate change significantly influences the concentration of airborne pollen, with elevated temperatures correlating with increased pollen concentrations in atmosphere (Cristofolini et al., 2024). Consequently, artificial pollination has become essential in kiwifruit production, as it directly enhances fruit set and quality (Antunes et al., 2022; Pozo et al., 2018). There exists a significant linear correlation between seed number and fruit weight in kiwifruit, with a requirement of approximately 850 seeds to produce a fruit weighing over 100 grams (Giannia et al., 2016; Sun et al., 2025).

Building on its widespread industrial and commercial applications in the latter half of the 20th century (Law, 2001), electrostatic technology has since been adopted in agriculture to improve microparticle deposition efficiency (Laryea and No, 2003). Its application is particularly advanced in pesticide spraying, where electrostatic forces have been shown to direct droplet trajectories toward the spray axis, significantly enhancing deposition (Abdel-Salam et al., 1995). In conventional pesticide spraying, only a small fraction of pesticide is retained on crops, while the remainder may result in environmental pollution. The integration of electrostatic technology has significantly improved deposition efficiency on crop leaves (Asano, 1986). In artificial pollination, Israeli researchers have achieved notable advancements in pneumatic-electrostatic pollination systems, developing a corona charging system suitable for dry pollen suspensions (Gan-Mor et al., 2009) and an induction charging system for liquid pollen (Law et al., 2000; 2001). To explore the effect of electrostatic charging on pollen dispersal and deposition, metallic replicas of almond flowers were constructed, and pollen dispersal experiments were conducted using both electrostatically charged and uncharged almond pollen, results showed that electrostatic charging significantly enhanced pollen deposition on floral surfaces. Furthermore, while uncharged pollen was uniformly distributed across the entire flower, charged pollen predominantly accumulated on the distal ends of the corolla and stigma (Vaknin et al., 2001a; Vaknin et al., 2001b). The impact of electrostatic-assisted pollination on yield of almond, kiwi, date, and pistachio was investigated, for almonds the average yield per tree increased by 13%, albeit with a slight reduction in nut size; in kiwifruit, seed count increased by 13.2%; for dates, fruit set rate in dates doubled while pollen usage was significantly reduced; in pistachio, the yield per plant increased by 12.7% alongside a 28% improvement in dehiscence rate (Gan-Mor et al., 2003). Focusing specifically on almond, subsequent research confirmed that electrostatic pollination enhanced yield compared to open pollination, despite a marginal decrease in nut weight. Eventually, electrostatic pollination can be used as a crucial supplementary method for pollen application in almond orchards when bee pollination is insufficient (Vaknin et al., 2001b). More importantly, physiological effects of electrostatic fields on crops have been discovered (Sidaway, 1966), as early as the 1960s, when placing Avena sativa in a unipolar environment of either charge resulted in significant growth stimulation (Krueger et al., 1962). Electrostatic technology is also beneficial to seeds: electric fields significantly promoted grain growth in wheat, optimize nutritional composition, and notably enhance germination length of wheat seeds (Ahmed et al., 2020; Chenah et al., 2024). Electrostatic treatment of pollen and seeds exerts a lasting effect on crop development (Dannehl, 2018), triggering a series of biological responses following pollination (Boussetta et al., 2012). The applied current enhances ion permeability in cell membranes, thereby triggering signal transduction and activating metabolic processes within the plant (Zhang and Hashinaga, 1997), ultimately leading to changes in fruit quality (Lander et al., 2025).

From aforementioned research findings, it is evident that electrostatic pollination shows advantages in enhancing pollen deposition on target surfaces, reducing pollen consumption while simultaneously increasing fruit yield. However, the impact of electrostatic pollination on physiological development processes and fruit quality of crops warrants further investigation. This study focuses on the electrostatic treatment of kiwifruit pollen and investigates the effects on entire physiological cycle of kiwifruit, encompassing pollen viability, pollen tube behavior, yield, fruit quality, and aroma. Indicators of kiwifruit quality were detected, including soluble solids content (SSC), sugar content (fructose, glucose, and sucrose), titratable acidity (TA), dry matter content (DM), vitamin C (VC), volatile organic compounds (VOCs), weight and seed count. This research provides a novel approach for future cultivation management practices in commercial kiwifruit orchards.

## 2 Materials and methods

### 2.1 *In vitro* germination of kiwifruit pollen

In *in vitro* pollen germination assays, a pollen grain is typically considered viable and capable of germination if length of its pollen tube exceeds the diameter of pollen grain (Sahar and Spiegel-Roy, 1984). Plant surfaces typically carry a weak negative charge in sunny conditions (Hunting et al., 2021), and electrostatically charged pollen has been shown to achieve greater deposition rates on flowers (Law et al., 2000). This enhanced deposition is attributed to electrostatic forces, which provide additional adhesion between pollen grains and stigma (Li et al., 2024). Here, pollen was charged by corona charging, the principle is illustrated in **Supplementary Figure 1**, where high voltage generates an ionized field around the sharp electrode (Zhao et al., 2024). Any pollen grains passing through this field are bombarded by ions, resulting in acquisition of electric charge (Dai and Law, 1995).

Building upon numerous beneficial effects of electrostatics on crops identified in existing research, this study designed a precision electrostatic pollinator (**Supplementary Figure 2a**), comprising powder-feeding motor, powder tank, corona needle, pipeline, and fan. The powder-feeding motor drives the rotation of a brush, and by adjusting motor voltage, the pollen output can be precisely controlled to either 1 mg or 2 mg per cycle, fan at the base serves as primary power source of pollinator, propelling pollen through an electrostatic field to reach flowers.

To evaluate the influence of electrostatic treatment on pollen viability and validate the beneficial effects of electrostatics on pollen germination, an electrostatic charging platform for pollinator was designed (**Supplementary Figure 2b**). Electrostatic pollinator was fixed on a stand, fan voltage controlled by an adjustable power supply, thereby regulating the airflow velocity within pollinator, while a control system (Arduino Mega 2560, L298N) managed the rotational speed of powder-feeding motor, controlling the pollen input rate. A high-voltage electrostatic generator (HC-60KV, Zhaoqing Hechuang Technology Co., Ltd) with a voltage range of 3-48.6 kV was employed. The device exhibited voltage instability below 10 kV, so the initial voltage was set at 10 kV, with increments of 2 kV, and termination voltage at 40 kV (10, 12, 14, 16…40 kV). To determine the enhancement effect of electrostatic treatment on pollen germination, a control group (CK) without electrostatic treatment was established.

Adjusting the knob of electrostatic generator to set voltage values, pollen electrostatically treated with different voltages was collected from pollen collection piece. Steps for germination experiment were as follows: prepare liquid culture medium by dissolving 10 g of sucrose and 0.01 g of boric acid in 100 mL of distilled water, heating the solution until it is completely dissolved (Božič and Šiber, 2022; Suanno et al., 2023); for each pollen sample, a small amount was transferred using a spatula into a centrifuge tube containing 7–8 mL of liquid culture medium; then centrifuge tubes were placed in a constant temperature shaker at 25°C and 100 rpm, after 3 hours of incubation, centrifuge tubes were removed; using a rubber-tipped dropper, a uniformly mixed droplet was aspirated from centrifuge tube and placed onto a glass slide. A coverslip was then applied, and slide was observed and photographed under an optical microscope (Olympus BX51 microscope, Japan). This procedure was repeated three times, with five fields of view captured each time, ensuring that each field contained at least 30 pollen grains.

### 2.2 Pollination experiment

Pollination experiments were conducted at the kiwifruit experimental station of Northwest A&F University, Shaanxi, China. Station is located at an altitude of 456 meters, with coordinates of 34°07’24.02’’ N latitude and 107°59’48.48’’ E longitude. The region experiences a temperate continental monsoon climate, characterized by an average annual temperature of 12.9 °C, an average annual precipitation of 609.5 mm, an average annual sunshine duration of 2015.2 hours, and an average annual frost-free period of 218 days.

Between late April and early May 2024, morphological characteristics of female Xuxiang kiwifruit flowers at various flowering stages were photographed and categorized, resulting in the classification of eight distinct flowering stages (**Supplementary Figure 3**). The kiwifruit experimental station cultivated male and female plants in a ratio of 1:6, with a substantial presence of artificially managed bee colonies in vicinity, to mitigate interference from wind pollination and bee pollination during artificial pollination (**Supplementary Figure 4a**), bagging experiments were conducted on April 28, 2024, prior to pollination. Flower buds close to blooming (**Supplementary Figure 1c**, **Figure 3b**) were selected and bagged. The receptivity of female kiwifruit plants peaks within one to two days after flowering. On May 7, 2024, the bags were removed, and fully opened flowers were inspected and marked (**Supplementary Figures 3e, f**). Artificial pollination was conducted at 10:00 AM on May 7, 2024, with the daily minimum temperature recorded at 14 °C and the maximum temperature at 28 °C (**Supplementary Figure 4b**).

**Figure 1:**
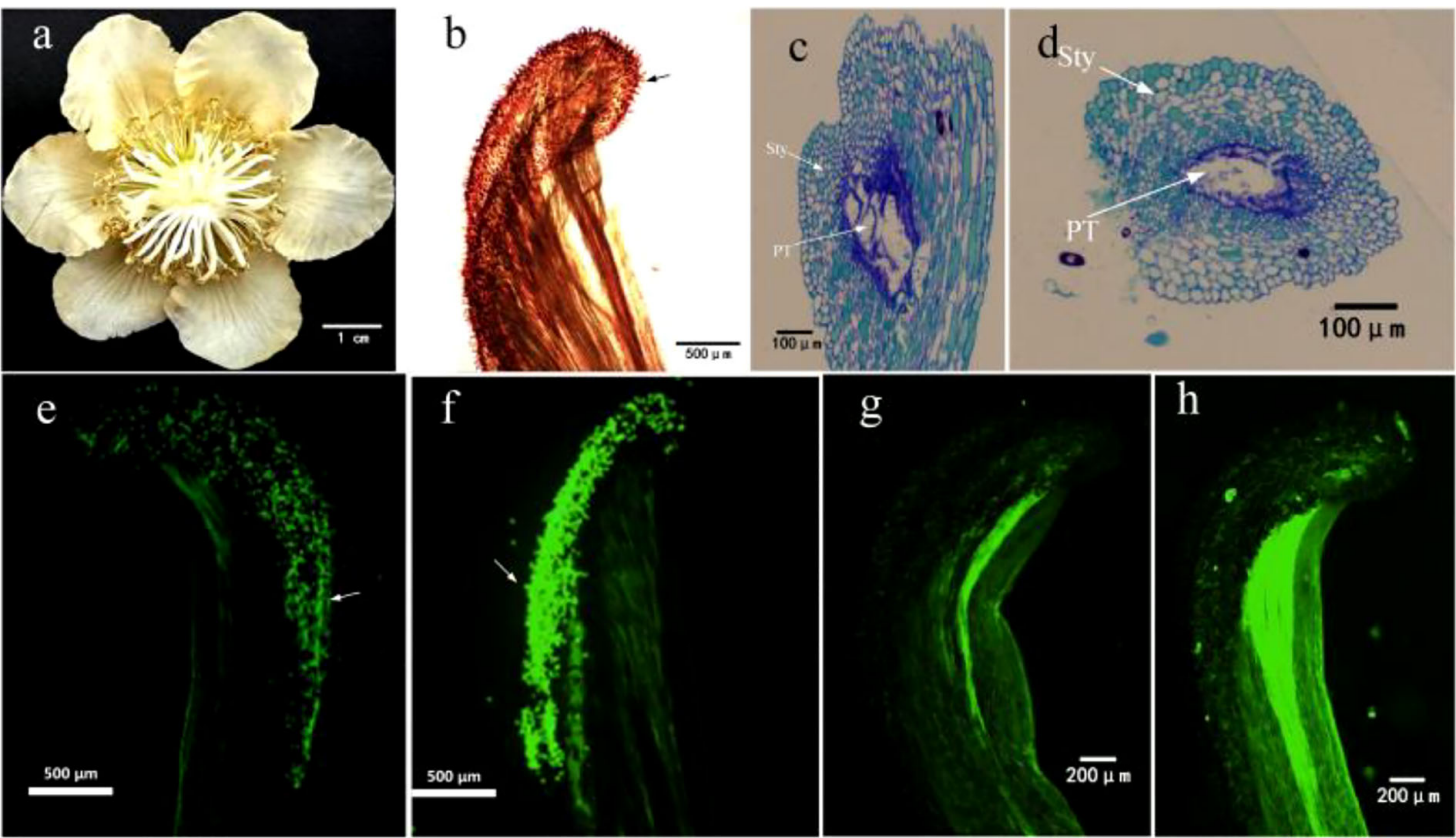
Morphologies of Xuxiang kiwifruit female flower **(a)** and stigmatic papillae **(b)**. Optical microscopic images of longitudinal **(c)** and cross **(d)** sections of stigma. Germination of pollen on the stigmatic surface with non-electrostatic treatment **(e)** and electrostatic treatment **(f)**. Pollen tube growth in style of non-electrostatic treatment **(g)** and electrostatic treatment **(h)**. Sty, style; PT, pollen tube.

In selecting electrostatic voltages, results from the *in vitro* pollen germination experiment were considered. Pollen germination rates and pollen tube lengths exhibited optimal performance at electrostatic voltages of 14 kV, 20 kV, 28 kV, 32 kV, and 38 kV, these voltages were chosen for electrostatic treatment of pollen in field experiment, with 0 kV serving as CK. Six Xuxiang kiwifruit trees of consistent age and growth vigor were selected. For each tree, flowers on branches located in the eastern, southern, western, and northern directions of the middle outer canopy were chosen for pollination. Kiwifruit pollen is light yellow, making it difficult to visually confirm successful adhesion to the stigma. Therefore, pollen was mixed with three times its weight in lycopodium powder. Successful pollination was indicated by a distinct red coloration of stigma (**Supplementary Figure 4d**).

### 2.3 Sample collection

The fruit size of Xuxiang kiwifruit exhibits a significant correlation with success of pollination and fertilization. It is imperative to conduct morphological observations of pollination, fertilization, and embryonic development closely associated with fruit set in Xuxiang kiwifruit. From the perspective of reproductive biology, this study aims to investigate the mechanisms by which electrostatically treated pollen influences fertilization and fruit quality in Xuxiang kiwifruit.

#### 2.3.1 Style collection and observation

At 30-minute intervals following artificial pollination, five flowers were sampled until 15 hours post-pollination. Using a blade, styles (including stigmas) were excised at the junction between ovary and style, with two styles collected from each flower. The samples were immediately fixed in 70% FAA (Formalin-Aceto-Alcohol) fixative solution. Pollen tube fluorescence observation was conducted using staining and squashing techniques. Staining procedure: styles were removed from fixative solution and rinsed three times with distilled water, each rinse lasting 5 minutes; then, styles were subjected to a softening treatment by immersion in 2 mol L^−1^ NaOH and incubated at 65 °C in a constant temperature oven for 3 hours; following this, samples were stained for 3 hours using a 0.1% aniline blue staining solution prepared in 0.1 mol L^−1^ K_3_PO_4_ solution. Squashing procedure: using forceps, a single style was gently transferred onto a glass slide containing a droplet of stain, due to softening treatment, styles became fragile, necessitating extreme care during handling; a coverslip was then placed over the style and gently pressed. Due to gradual attenuation of fluorescence intensity over time, observation and capturing of the image under fluorescence microscope (Olympus BX53, Japan) was done shortly after its preparation.

#### 2.3.2 Fruit collection and classification

Firmness was utilized as an indicator of maturity in this study, with the fruit harvest period defined as Phase 0 (P0), then kiwifruits were allowed to naturally soften at room temperature (25 °C), when the firmness reached a range of 5–8 N, this stage was designated as Phase 1 (P1). Fruit firmness was measured using an electronic penetrometer (Willowbank Electronics Ltd., Napier, New Zealand). During P0, flesh firmness ranged between 100–110 N, with SSC exceeding 6.5% and dry matter content of 18.7%. For each test group, 30 fruits of similar size and shape were selected, and fruits were randomly harvested from different trees and branches to ensure representative sampling. It should be noted that, to ensure data consistency, after measuring firmness to determine fruit maturity, the samples were chopped, flash-frozen in liquid nitrogen, and stored in a -80 °C freezer. This protocol was implemented to facilitate subsequent analyses of sugar content, TA, and VOCs in flesh.

### 2.4 Determination of SSC, TA, DM and VC

After measuring firmness, kiwifruit samples were homogenized into juice. SSC was measured using a digital pocket refractometer (PAL-1, Atago Co., Ltd., Japan), with the results expressed as a percentage of sugar content.

Contents of fructose, glucose, and sucrose were determined using commercial assay kits, specifically Solarbio BC2455, BC2505, and BC2465, respectively. Following the manufacturer’s instructions, the concentrations of all three sugars were expressed on a fresh weight basis, with units of g kg^-1^.

TA was determined using the method described by Xu (Xu et al., 2019), with the concentration expressed as a percentage of citric acid. One gram of kiwifruit peel was diluted in 3 mL of distilled water and filtered through a 0.22 μm pore-size membrane. Subsequently, 3 mL of the filtrate was titrated with 0.1 mol L^-1^ sodium hydroxide.

DM is defined as the ratio of dry weight to fresh weight. The kiwifruit was sliced along the central axis, weighed, and the initial weight recorded, slices were then placed in drying oven for over 24 hours, after which they were reweighed and the final weight recorded.

VC was conducted as follows, the flesh was homogenized into juice, and 3 mL of the juice was promptly transferred into a conical flask containing 3 mL of a 2% oxalic acid solution, the solution was then titrated with a standardized 2,6-dichlorophenolindophenol solution until a pink color persisted for 15 seconds, a blank control was simultaneously performed for comparison.

### 2.5 Detection of VOCs

Detection of VOCs is a critical approach for analyzing aroma components and flavor characteristics of fruits. During P1, kiwifruit develops a distinctive aroma. Xuxiang, a cultivar of green-fleshed Actinidia chinensis var. deliciosa, exhibits a fragrance reminiscent of citrus and melon (Garcia et al., 2012). VOCs were detected by gas chromatography-mass spectrometry (GC-MS) system (Agilent 5977B, Agilent Technologies, Inc., USA). With electronic balance, 1 g of fruit peel was weighed and transferred into a 40 mL headspace vial pre-cooled with liquid nitrogen, 2 mL of saturated saline solution, and 5 μL of internal standard (3-nonanone/MTBE = 1:6000, v/v), a magnetic stir bar. After sealing, vial was placed on magnetic stirrer and equilibrated at 40 °C for 10 minutes. Subsequently, aged extraction fiber (DVB/CAR/PDMS; Supelco, Inc., Bellefonte, PA, USA) was inserted into headspace vial for 30-minute extraction, fiber was then introduced into injection port of Agilent 7890B gas chromatograph (Agilent Technologies, Palo Alto, CA, USA), desorbed at 230 °C for 3 minutes to release VOCs, then analyzed by Agilent 5875C mass selective detector (Agilent Technologies). Initial temperature of GC column oven was 40 °C and held for 3 min, then increased to 100 °C at a rate of 3 °C min-1, and then further raised to 245 °C at a rate of 5 °C min^-1^. Carrier gas was helium with a purity of 99.999%. Mass spectra (MS) conditions: electron ionization (EI) mode at 70 eV, mass scan range 35–350 m/z. VOCs was qualitatively analyzed with chromatograms from GC-MS by Qualitative Analysis B.07.00, identified based on NIST 2014 MS library and validated by retention indices.

### 2.6 Statistical analysis

The statistical significance of differences between mean values was determined using Student’s t-tests or ANOVA with Tukey’s multiple comparison test. Different asterisks against error bars of histograms are used to indicate means that are statistically different at P < 0.05.

## 3 Results and discussion

### 3.1 Impact of electrostatic treatment on pollen viability

**Figure 2:**
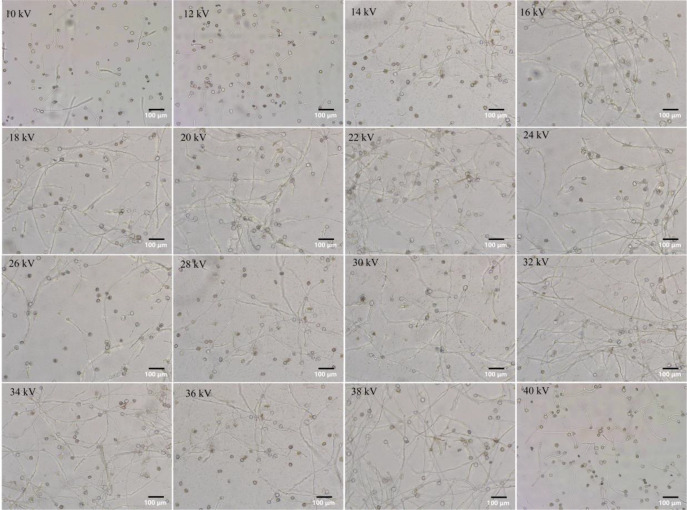
Pollen germination and pollen tube growth by electrostatic treatment with various voltages. Scale bar: 100 μm.

**Figure 3:**
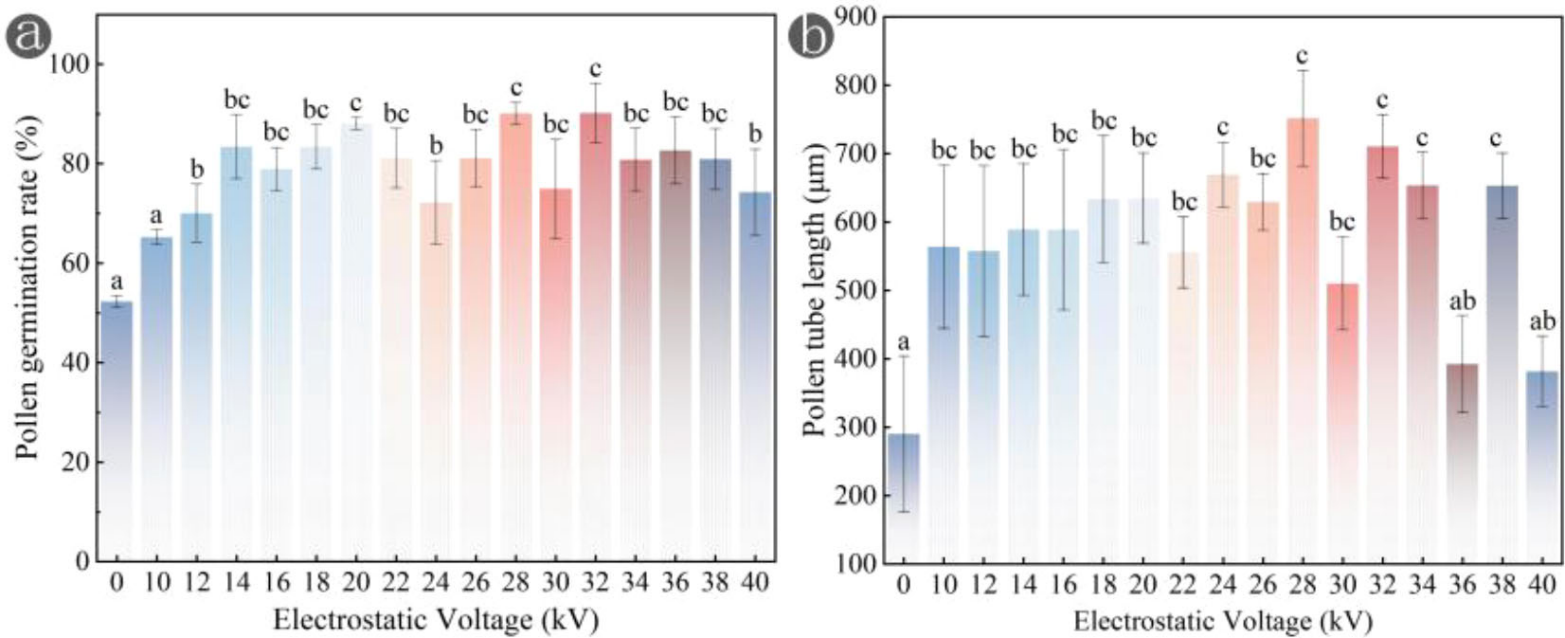
Statistics of pollen germination rate **(a)** and pollen tube length **(b)** under electrostatic treatment with various voltages. Statistical differences were calculated by one-way ANOVA. Different letters indicate statistically significant differences determined by Tukey’s multiple comparison test (P < 0.05).

Representative images were selected for analysis, kiwifruit pollen tubes exhibited a translucent appearance. **Supplementary Figure 5** illustrates pollen germination without electrostatic treatment, showing that approximately half of pollen grains in the field of view were germinated. It is noteworthy that the performance of *in vitro* pollen germination is significantly influenced by composition of culture medium, such as the ratios of sucrose and boric acid (Su et al., 2024), as well as by endogenous hormones of pollen itself (Calabrese and Agathokleous, 2021; Tunur and Çetinbaş-Genç, 2024). This study aims to analyze impact of electrostatics on pollen germination under ambient temperature, therefore, interference of other factors was not further investigated. As illustrated in **Figure 2**, the number of germinated pollen grains significantly increased following electrostatic treatment, and pollen tube length also exceeded that of CK. Further statistical analysis was conducted on germination rates of pollen treated with different voltages (**Figure 3a**). It was found that under the *in vitro* culture conditions employed in this study, CK exhibited a germination rate of 52.32%, indicating that the pollen already possessed relatively high viability. Germination rate of pollen treated with 10 kV electrostatic voltage was 65.31%, when electrostatic voltage reached 32 kV, the germination rate peaked at 90.22%, representing a 37.9% increase compared to CK. Within the electrostatic voltage range of 10–40 kV, germination rate of pollen grains consistently achieved a desirable enhancement effect (>52.32% in CK). In this research, pollen germination rates were consistently promoted under various voltage, however, pollen tube growth exhibited suboptimal performance at higher voltages, which suggests that the physiological effects of electrostatic fields on plants are not always positive, the finding aligns with Murr’s research: high-intensity electric fields inhibited the growth of grass seedlings (Murr, 1963; 1964).

In CK, average length of pollen tubes was 289.98 μm, following electrostatic treatment, the length significantly increased, at a voltage of 10 kV, length reached 563.82 μm; when electrostatic voltage was 28 kV, the length peaked at 751.69 μm, representing a 159.22% increase compared to CK. Electrostatic treatment can promote growth of pollen tubes (**Figure 3b**). However, at electrostatic voltages of 36 kV and 40 kV, the growth performance of pollen tubes was suboptimal, with lengths of 392.65 μm and 381.61 μm, respectively. Nevertheless, these lengths still exceeded those observed in CK.

### 3.2 Pollen tube behavior in styles

In botany field, fluorescence microscopy is particularly well-suited for observing the growth of pollen tubes as they penetrate style and enter ovary (Martin, 1959). In numerous crop species, callose is abundantly present in pollen tube walls but is relatively scarce in surrounding stylar tissues. Callose exhibits selective affinity for aniline blue, enabling it to emit distinct green fluorescence under ultraviolet illumination, which contrasts sharply with the dark background. This principle was utilized in the study to observe growth dynamics of pollen tubes within styles of Xuxiang kiwifruit. The styles of Xuxiang kiwifruit exhibit a milky white coloration (**Figure 1a**), and stigmatic surface is densely covered with papillae (**Figure 1b**), following pollen germination, pollen tubes penetrate the papillae surface and enter style (Hiscock et al., 2002); longitudinal (**Figure 1c**) and transverse (**Figure 1d**) sections of stigma reveal that the style possesses a hollow structure. Kiwifruit pollen can germinate on stigmatic surface within 1 hour after landing. Observations under a fluorescence microscope revealed that the number of fluorescent spots on stigmatic surface subjected to electrostatic pollination (**Figure 1f**) was significantly higher compared to that on the stigma without electrostatic treatment (**Figure 1e**). 3 hours after pollination, pollen tubes were observed extending downward through style. In styles subjected to electrostatic pollination (**Figure 1h**), pollen tubes exhibited faster growth rates, and the number of pollen tubes was significantly greater compared to those in styles without electrostatic treatment (**Figure 1g**). To this point, it is evident that electrostatic treatment demonstrates a pronounced beneficial effect on both pollen germination on stigmatic surface and subsequent growth of pollen tubes within style.

**Figure 4:**
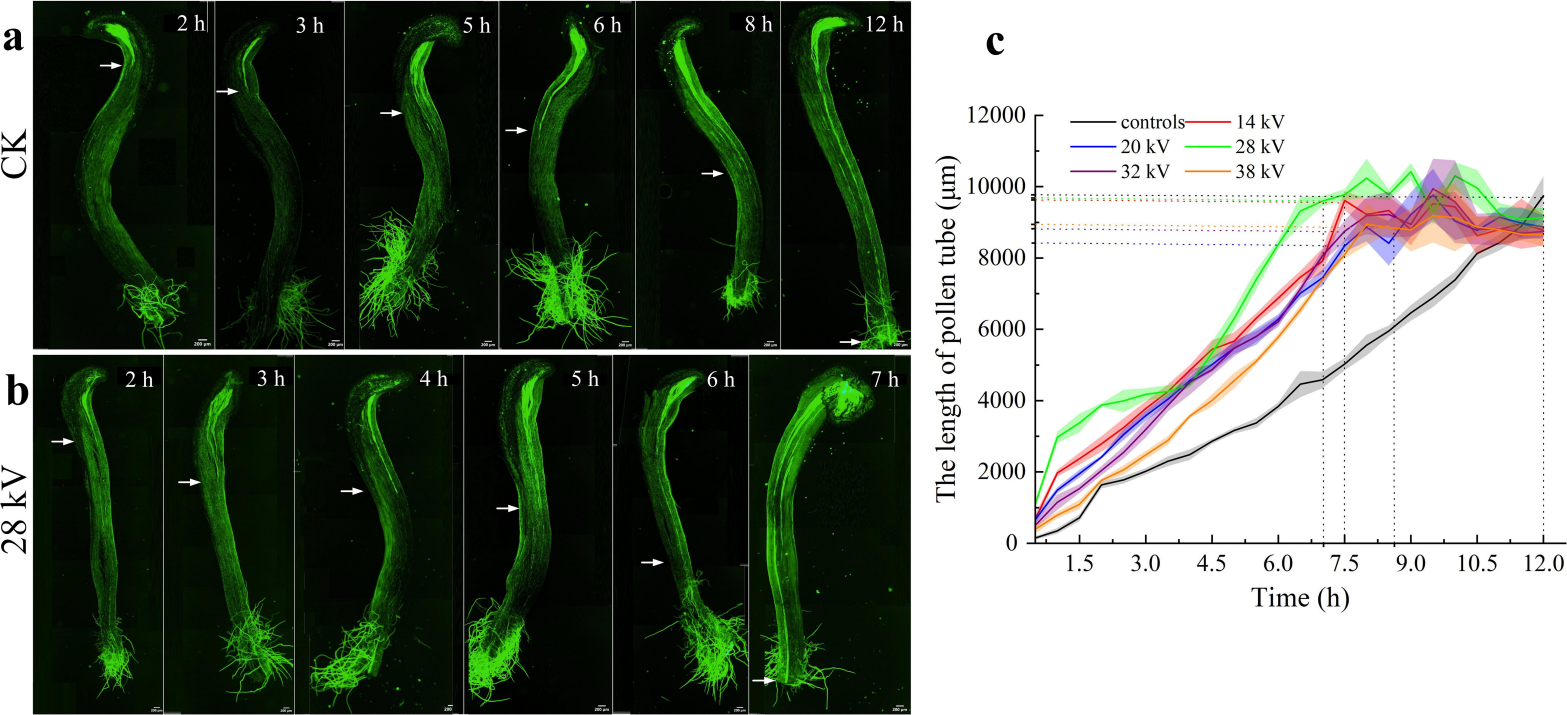
Fluorescence observation of pollen tube growth process. Pollen tube growth in styles of Xuxiang kiwifruit cultivars, pollen tube length from left to right (from early to later pollination stage), including 6 time points: 2 h, 3 h, 5 h, 6 h, 8 h, 12 h: **(a)** after artificial pollination without electrostatic treatment; **(b)** after artificial pollination with electrostatic treatment, the scale bar is 200 μm. **(c)** The length of pollen tubes in styles with electrostatically untreated and treated pollen (20, 28, 32 and 38 kV, respectively).

Stigma of kiwifruit is evidently well-suited for pollen germination, within 1 hour after pollination, pollen grains begin to germinate on stigmatic surface and subsequently penetrate through stigma interstices to enter stylar tissue. 3 hours after non-electrostatic treatment, the average length of pollen tubes in *in vitro* germination was measured to be 289.98 μm, while length of pollen tubes originating from pollen grains germinated on stigmatic surface was 716.94 μm; consistent with results observed in *in vitro* germination, electrostatic treatment significantly promoted the growth of pollen tubes, 3 hours after electrostatic pollination (28 kV), average length reached 3376.95 μm. The enhancing effect of electrostatic treatment on pollen tube growth was particularly pronounced within stylar tissue, for instance, at 3-hour mark, length of pollen tubes from electrostatic pollination was 4.71 times greater than that of pollen tubes from non-electrostatic pollination. As illustrated in **Figure 4** and **Supplementary Figure 6**, pollen subjected to electrostatic treatment (regardless of the voltage applied), consistently exhibited longer pollen tube growth compared to CK (black line in **Figure 4c**). Statistical analysis revealed that the majority of Xuxiang kiwifruit styles measured between 7 and 9 cm in length. Under electrostatic pollination, pollen tubes reached style base and began entering the ovary within 7 hours (**Figure 4b**), in contrast, under non-electrostatic pollination, pollen tubes required more than 12 hours to grow to style base (**Figure 4a**). These findings provide compelling evidence to suggest that electrostatic treatment significantly enhances pollination effect and fertilization processes in kiwifruit. A potential explanation is that the electric field regulates a series of cellular activities, including release of extracellular DNA, proteins, and intercellular polysaccharide adhesives (Calabrese and Agathokleous, 2021).

### 3.3 Fruit weight and seed count

**Figure 5:**
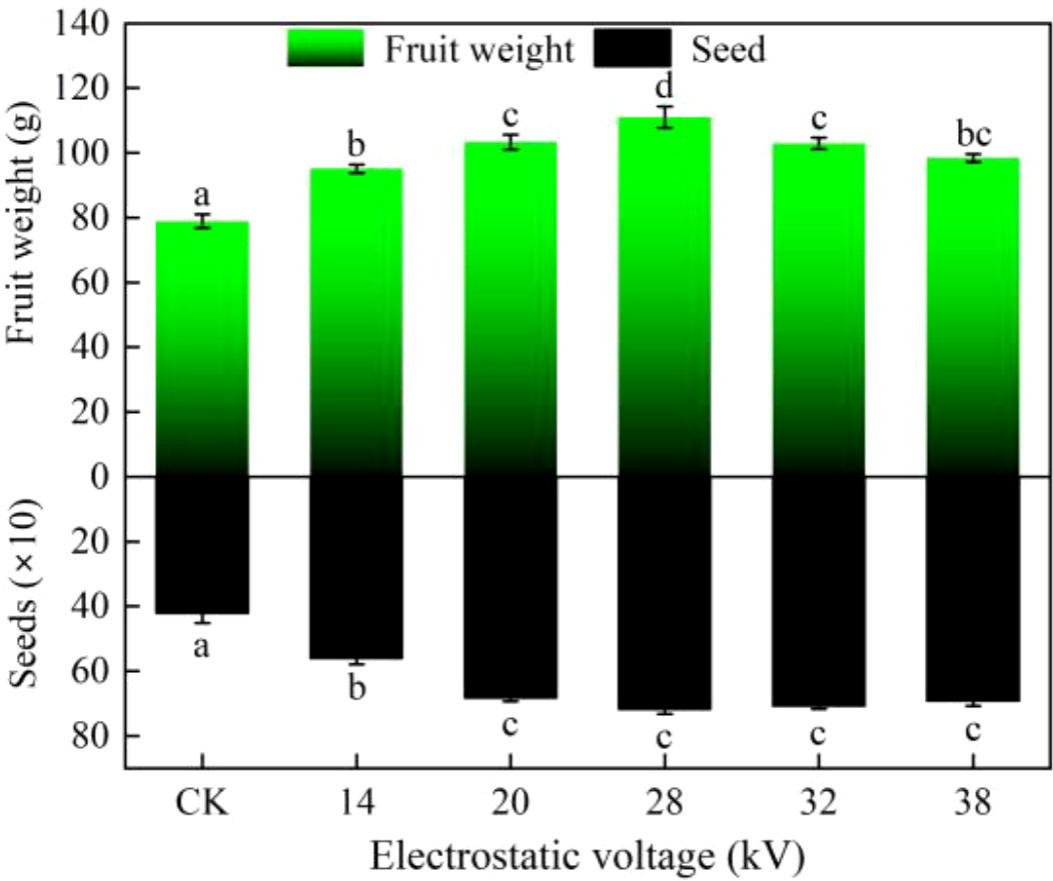
Statistical analysis of fruit weight and seed count in Xuxiang kiwifruit. Statistical differences were calculated by one-way ANOVA. Different letters indicate statistically significant differences determined by Tukey’s multiple comparison test (P<0.05).

Electrostatic treatment has been shown to exert lasting effects on plant growth (Dannehl, 2018). In kiwifruit, electrostatic pollination significantly increases both seed number and yield, numerous studies have documented notable yield-enhancing effects of electrostatic pollination (Gan-Mor et al., 2003; Maheshwari and Grewal, 2009). It should be noted that all kiwifruit in this research were treated without growth enhancer and cultivated under identical water and nutrient conditions until P0. Fruits subjected to non-electrostatic pollination exhibited an average weight of approximately 80 g and contained around 420 seeds, which are within the normal range. One can see in **Figure 5**, electrostatic pollination significantly increased the average fruit weight, at 28 kV, fruit weight increased by around 30 g, and seed count was 1.7 times higher compared to CK. When electrostatic voltage was increased to 32 kV and 38 kV, a slight decline in fruit weight and seed count was observed; however, a consistent enhancement effect was still evident.

### 3.4 Impact of electrostatic pollination on fruit quality

**Figure 6:**
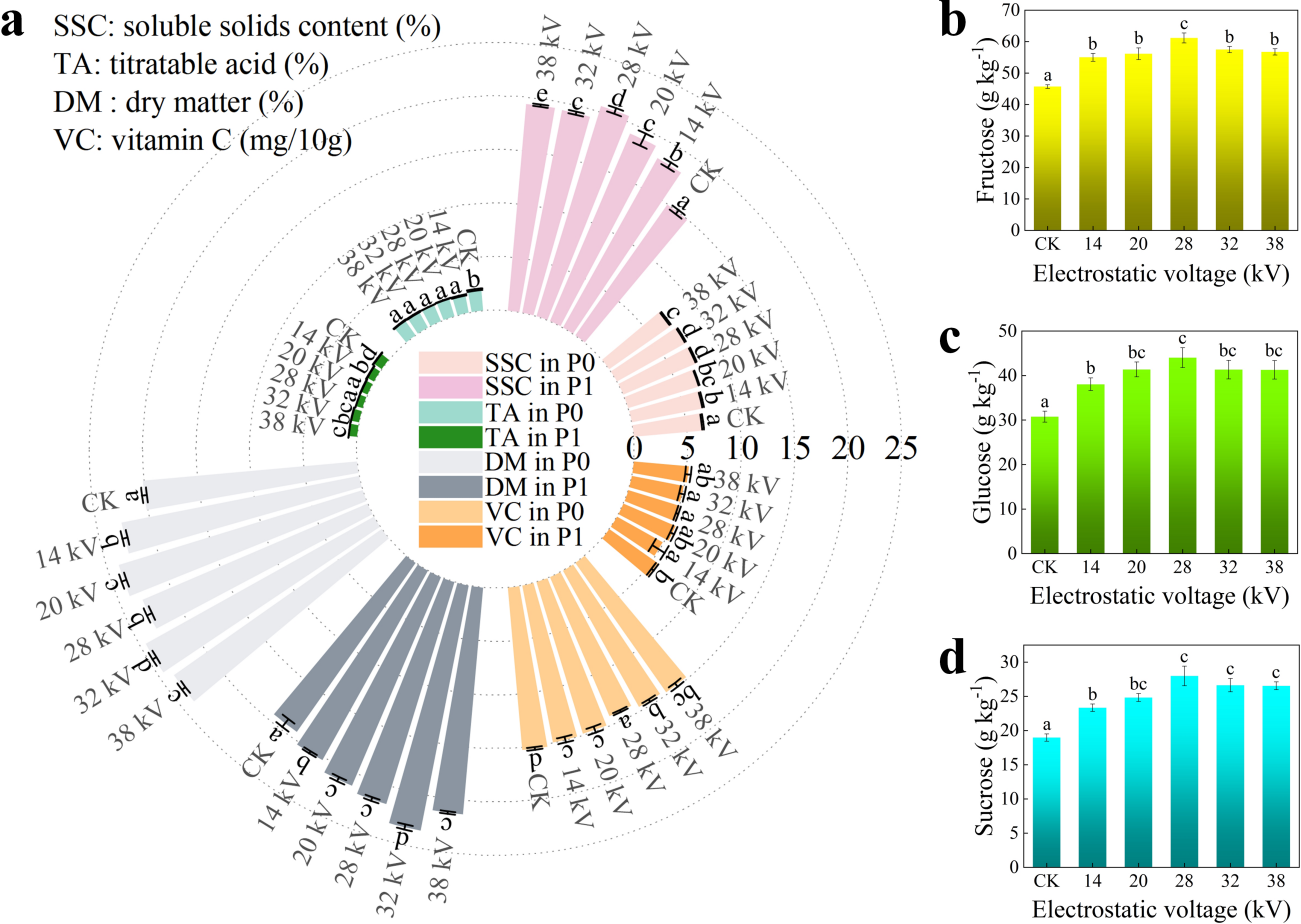
In picking and ripening period of Xuxiang, the content of each component of taste compounds under different electrostatic treatment with various voltages, **(a)** including SSC, TA, DM, and VC. **(b)** fructose content, **(c)** glucose content, **(d)** sucrose content. Statistical differences were calculated by one-way ANOVA. Different letters indicate statistically significant differences determined by Tukey’s multiple comparison test (P<0.05).

The determination of kiwifruit edibility can be assessed based on its firmness (Benelli et al., 2022). Previous studies have classified the maturity stages of ‘Hayward’ kiwifruit based on flesh firmness into under-ripe (7.8-11.8 N), ripe (4.9-7.7 N), and over-ripe (2.9-4.8 N) (Garcia et al., 2013). Stanley proposed that kiwifruit with a firmness below 9–13 N meets consumer preferences for texture (Stanley et al., 2007). At the time of harvest, kiwifruit must exhibit a firmness greater than 62 N, and SSC must reach 6.2-6.5% (Walsh et al., 2020). As shown in **Figures 6a**, in P0, electrostatic pollination significantly increased SSC compared to CK; in P1, SSC showed a substantial overall improvement, with the enhancement effect of electrostatic pollination being even more pronounced: at 28 kV, the SSC increased by 5.6%, which demonstrates that electrostatic pollination effectively enhances sugar content in Xuxiang kiwifruit. Similar to the treatment of maize seeds with HVEF, a 63.7% increase in soluble sugar content was observed (Lu et al., 2025). Contents of three sugar components in kiwifruit during P1 were tested (**Figures 6b–d**). Sugars are a major constituent of SSC, and their concentrations followed trends similar to those of SSC, varying with the applied voltage. Compared to CK, all three sugars exhibited significant increases to varying degrees. Overall, fructose was the most abundant sugar, reaching 61.16 g kg^-1^ at 28 kV (45.71 g kg^-1^ in CK). Glucose was the least abundant, with a concentration of 44.02 g kg^-1^ at 28 kV (30.78 g kg^-1^ in CK). Sucrose had the lowest concentration, measuring 27.98 g kg^-1^ at 28 kV (18.97 g kg^-1^ in CK).

During P0, kiwifruit exhibited a relatively high TA, electrostatic pollination reduced TA content, with the CK showing a TA of approximately 2%, while fruits subjected to electrostatic pollination had a TA of 1.7%. In P1, TA content decreased to some extent as the kiwifruit matured, and TA from electrostatic pollination was consistently lower than that of the control group. These results suggest that electrostatic pollination may contribute to a reduction in the acid of kiwifruit.

During P0, electrostatic pollination significantly increased DM content of kiwifruit flesh, at an electrostatic voltage of 32 kV, DM was elevated by 21.39%; in P1, DM decreased due to a series of biological changes, such as starch hydrolysis, However, the enhancing effect induced by electrostatic pollination remained significant, with DM reaching 23.34% at 32 kV (compared to 19.3% in CK).

Electrostatic pollination resulted in a slight reduction in VC content. During P0, the VC content in CK was 15.238 mg 10g^-1^, while at 28 kV, VC decreased to a minimum of 14.143 mg 10g^-1^. In P1, VC content further declined during fruit ripening due to enzymatic activity and oxidation reactions (Nishiyama et al., 2004). VC in kiwifruit subjected to electrostatic pollination was consistently lower than that in CK. These findings suggest that electrostatic pollination reduces VC content in kiwifruit. Based on comprehensive analysis of quality indicators, it can be concluded that electrostatic pollination enhances the sweetness and overall taste of Xuxiang Kiwifruit.

### 3.5 Impact of electrostatic pollination on VOCs

**Figure 7:**
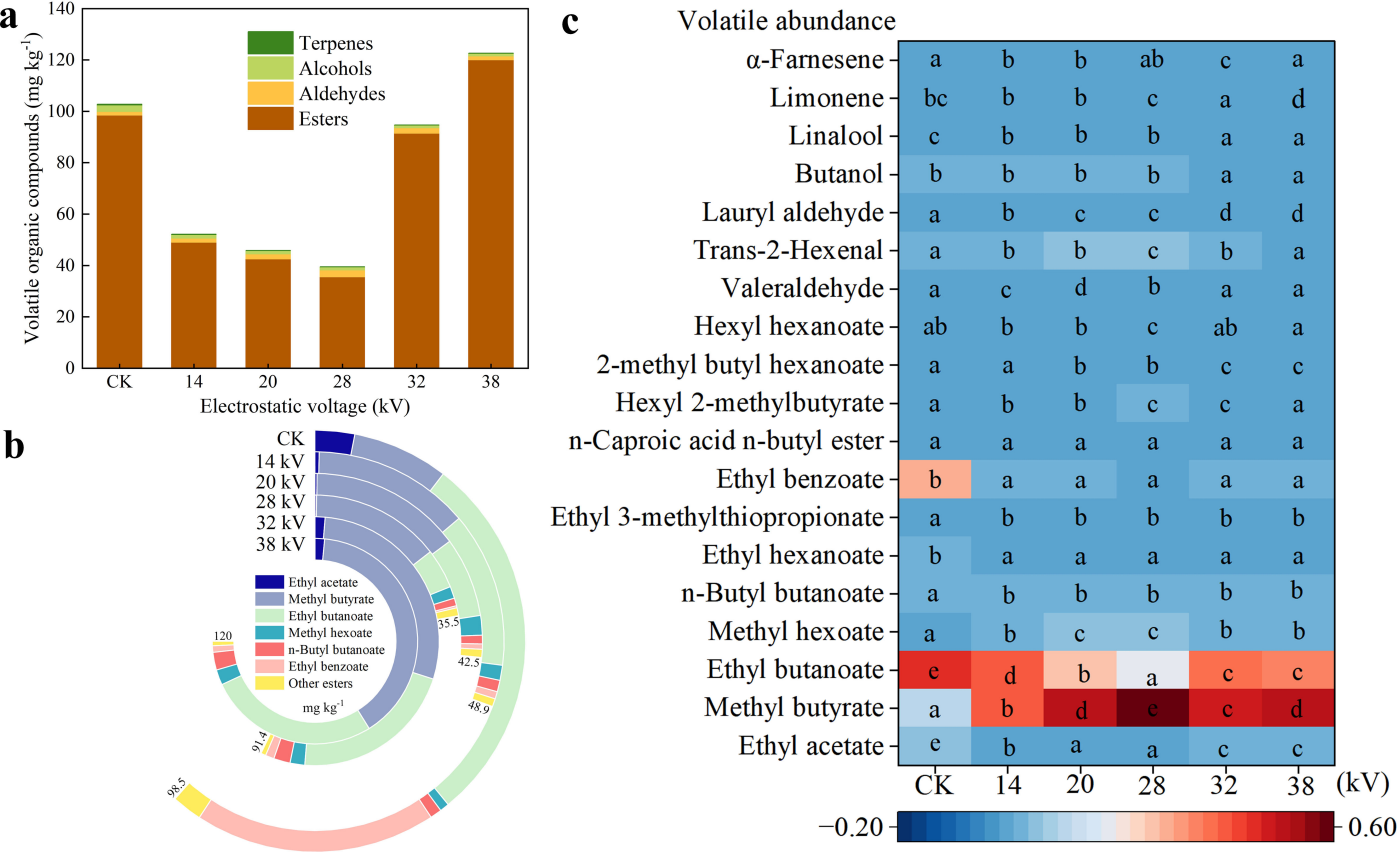
Volatile organic compounds in Xuxiang at ripening stage (P1) under electrostatic treatment with different voltages **(a)**; **(b)** content of esters under electrostatic treatment with different voltages. **(c)** Heatmap of each volatile organic compound content under electrostatic treatment with different voltages. Statistical differences were calculated by one-way ANOVA. Different letters indicate statistically significant differences determined by Tukey’s multiple comparison test (P < 0.05).

Kiwifruit aroma is affected by multiple volatile compounds, which significantly influence flavor and palatability. In P1, a total of 21 volatile organic compounds were detected in Xuxiang kiwifruit, including esters, aldehydes, alcohols, and terpenes. VOCs was lower than that in CK at 14 kV, 20 kV, and 28 kV, but increased at 38 kV (**Figure 7a**), esters were the most abundant, accounting for 95.86% of VOCs in CK, while contents of alcohols, aldehydes, and terpenes were relatively low. Therefore, a further analysis of ester compounds was conducted (**Figure 7b**), in CK, ethyl benzoate (29.65%) and ethyl butanoate (45.89%) were the most abundant. In fruits under electrostatic pollination, ethyl benzoate significantly decreased (from 0.35% to 1.66%). Notably, electrostatic treatment markedly increased methyl butyrate, reaching 45.77% at 32 kV and 63.41% at 38 kV (compared to 11.82% in CK). Trends in VOCs under different electrostatic voltages are presented more intuitively in heatmap (**Figure 7c**): due to color variations, it is evident that the ethyl benzoate in fruits under electrostatic pollination significantly decreased, while methyl butyrate exhibited a substantial multiplicative increase, peaking at 28 kV, which suggest that electrostatic pollination enhances kiwifruit flavor, imparting aromas reminiscent of apple and pineapple. However, based on the detected data, it is hypothesized that the beneficial effects of electrostatic pollination on aroma may become more pronounced at higher voltages.

## 4 Discussion

As a supplement to insufficient natural pollination, artificial pollination ensures high yields in orchards, previous studies have confirmed the natural compatibility between pollen and stigmas (Sun et al., 2025). Application of electrostatic technology in agriculture has yielded excellent results, its principle involves passing pollen, pesticides, or fertilizer sprays through an electrostatic field to charge particles, enhancing their adhesion and coverage on plant surfaces. More importantly, physiological effects of electrostatic fields on crops have been discovered (Sidaway, 1966), as early as the 1960s, Krueger observed that placing *Avena sativa* in a unipolar environment of either charge resulted in significant growth stimulation (Krueger et al., 1962). In the 21st century, Gan-Mor found that electrostatic pollination positively influences fruit quality and yield (Gan-Mor et al., 2003; 2009). Besides its effectiveness on particulate pollen germination, electrostatic technology also benefits seeds: electric fields significantly promoted grain growth in wheat, optimize nutritional composition, and notably enhance germination length of wheat seeds (Ahmed et al., 2020; Chenah et al., 2024). In this research, pollen germination rates were consistently promoted under various voltage, however, pollen tube growth exhibited suboptimal performance at higher voltages, which suggests that the physiological effects of electrostatic fields on plants are not always positive, the finding aligns with Murr’s research: high-intensity electric fields inhibited the growth of grass seedlings (Murr, 1963).

To explore the mechanism for higher fruit set rates in crops such as kiwifruit and date palms under electrostatic pollination, it is rational to hypothesize, electrostatic treatment enhances their fertilization and developmental processes. From sample observation, stigma provides an ideal environment for pollen germination, and pollen tube growth in style is highly adaptive. Under electrostatic pollination, pollen tubes reach the style base and release sperm cells earlier than under natural conditions. A potential explanation is that the electric field regulates a series of cellular activities, including release of extracellular DNA, proteins, and intercellular polysaccharide adhesives (Calabrese and Agathokleous, 2021). Furthermore, electrostatic treatment enhances fertilization, leading to earlier division of primary endosperm compared to natural conditions, recent studies have similarly reported that maize treated with high-voltage electrostatic field (HVEF) exhibits improved development (Lu et al., 2025). Electrostatic treatment has been shown to exert lasting effects on plant growth (Dannehl, 2018). In kiwifruit, electrostatic pollination significantly increases both seed number and yield, numerous studies have documented notable yield-enhancing effects of electrostatic pollination (Gan-Mor et al., 2003; Maheshwari and Grewal, 2009).

Excitingly, in assessment of fruit quality, electrostatic treatment was found to significantly increase SSC in kiwifruit, a similar trend was observed by Lu, treating maize seeds with HVEF resulted in a 63.7% increase in soluble sugar (Lu et al., 2025). SSC and TA are critical indicators of kiwifruit quality, electrostatic pollination resulted in lower TA content, and glucose, fructose, and sucrose were elevated to varying degrees alongside an increase in SSC. These findings suggest that kiwifruit under electrostatic pollination exhibit a sweeter taste profile. However, electrostatic treatment does not always exert beneficial effects, VC, an essential nutritional component of fruits, showed a reduction in content in this study; VOCs, which determine the aroma of kiwifruit, only at higher voltages did ester compounds significantly increase. Integrating previous research with the findings in this study, electrostatic pollination demonstrates significant beneficial effects on kiwifruit production. During the collection of fruit samples, it was also observed that kiwifruit subjected to electrostatic pollination had a shorter storage period. While fruits in CK maintained higher firmness, those from electrostatic pollination were already in edible state. The mechanisms underlying these changes induced by electrostatic treatment require further in-depth analysis from cytological and genetic perspectives.

## 5 Conclusions

Electrostatic pollination enhances pollen viability, consistently benefiting kiwifruit pollen in *in vitro* germination. At an electrostatic voltage of 32 kV, germination rate increased by 37.9%, while at 28 kV, pollen tube length increased by 159.22%. Stigmatic surface provides an optimal environment for pollen germination, and electrostatic treatment significantly enhances pollen germination rate on stigma. Pollen tube length under electrostatic pollination was 4.71 times that of non-electrostatic pollination, and they reached style base 5 hours earlier. And electrostatic pollination increased fruit weight by 37.5% and seed number by 70% (at 28 kV). Excitingly, in assessment of fruit quality, electrostatic treatment was found to significantly increase SSC kiwifruit, TA is also a critical indicator of kiwifruit quality, electrostatic pollination resulted in lower TA content, these findings suggest that kiwifruit under electrostatic pollination exhibit a sweeter taste profile. However, electrostatic treatment does not always exert beneficial effects, VC, an essential nutritional component of fruits, showed a reduction in content in this study; VOCs, which determine the aroma of kiwifruit, only at higher voltages did ester compounds significantly increase.

## Data Availability

The original contributions presented in the study are included in the article/**Supplementary Material**. Further inquiries can be directed to the corresponding author.
