# Supplementary material for: Electrostatic treatment enhances pollen viability and improves fruit quality in ‘Xuxiang’ kiwifruit
Source: Front Plant Sci. 2026 Mar 30;17:1769809. doi: 10.3389/fpls.2026.1769809 (PMC13070765; doi:10.3389/fpls.2026.1769809)
Supplement: Supplementary file 1 [file Supplementaryfile1.docx]

Supplementary Material

# Supplementary Figures


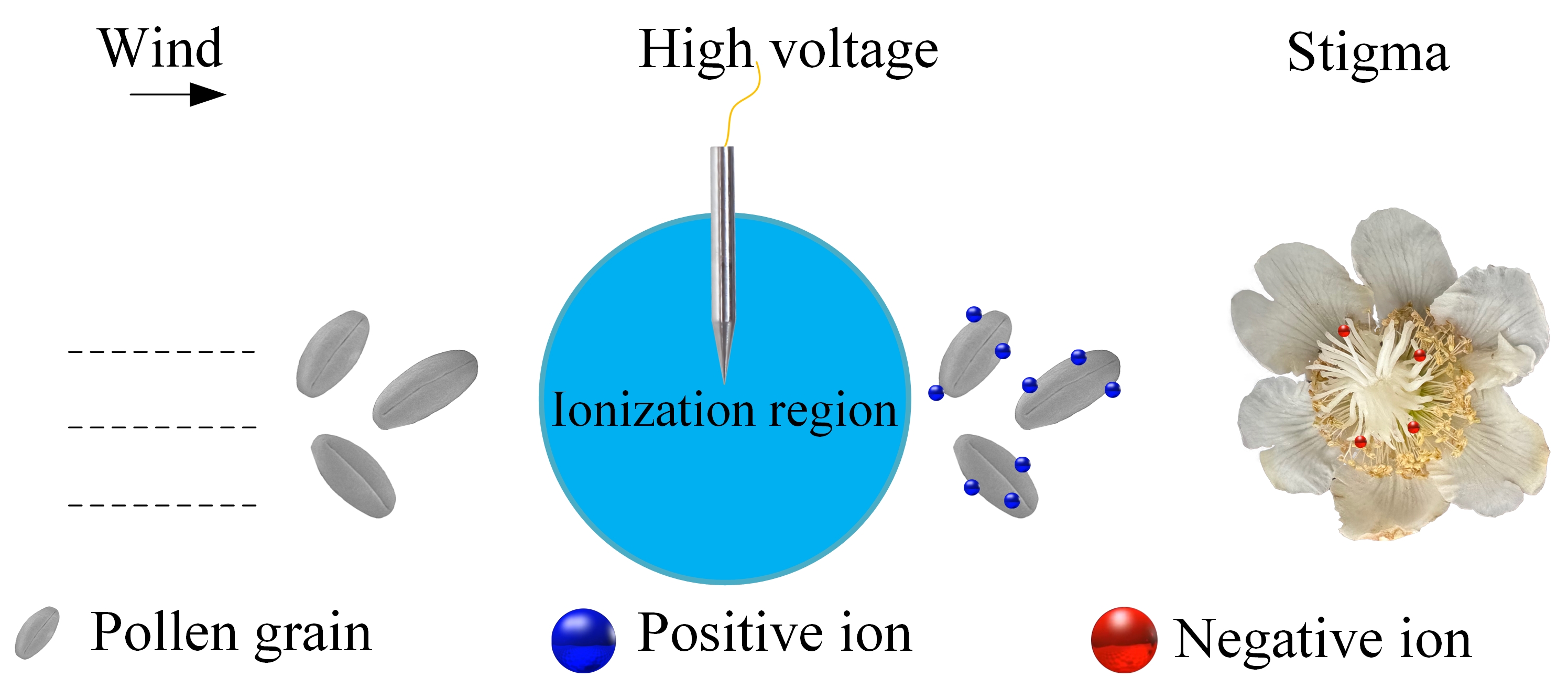


**Supplementary Figure 1.** Charge effect in pollen particle electrostatic delivery.


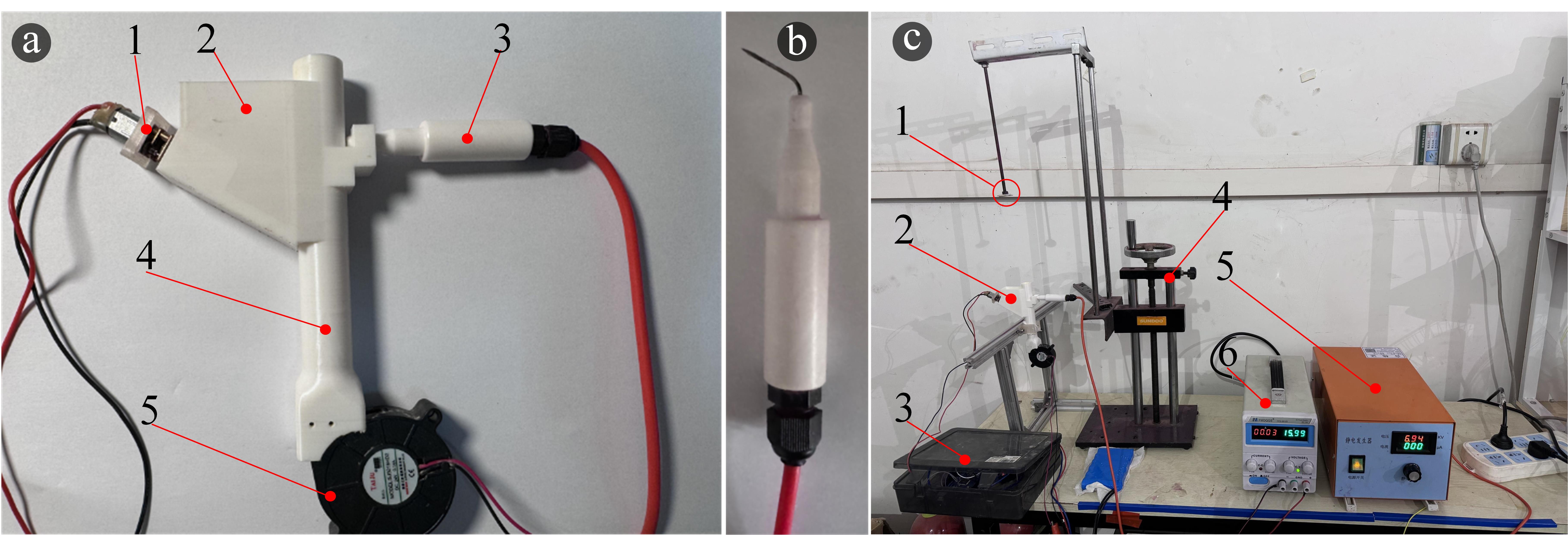


**Supplementary Figure 2.** Charge effect in pollen particle electrostatic deliveryStructure diagram of air-fed and electrostatic kiwifruit precision pollinating platform. (a) Electrostatic pollinator (1：powder-feeding motor; 2: powder tank; 3: Corona needle; 4: pipe; 5: fan). (b) Corona needle. c Pollen charging system (1: pollen collection piece; 2: electrostatic pollinator; 3: controller box; 4: lifter; 5: electrostatic generator; 6: adjustable power supply).


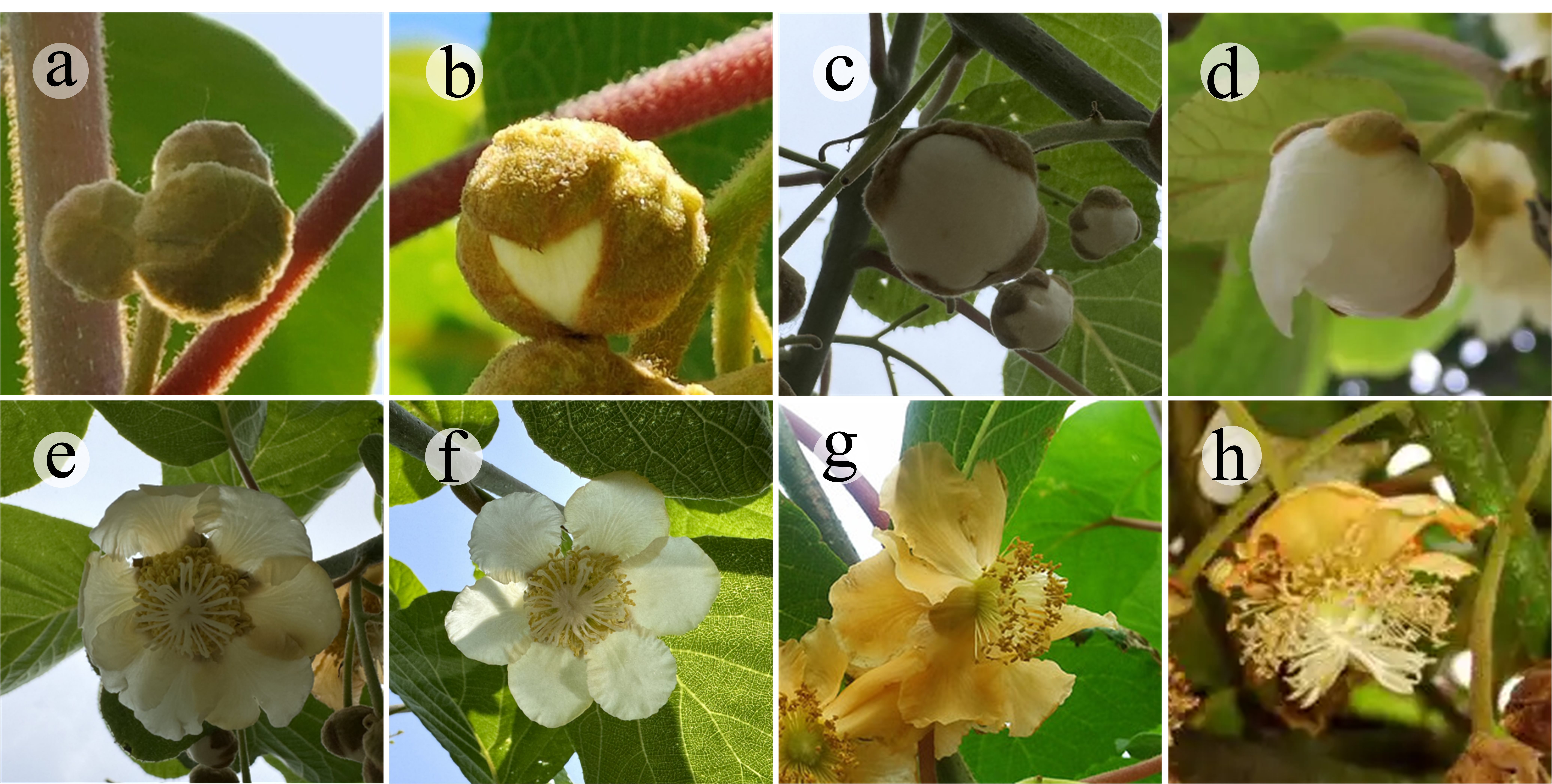


**Supplementary Figure 3.** Flower morphology of Xuxiang kiwifruit cultivar at different flowering stages. (a) Flower buds growing. (b) Sepals begin to separate. (c) Corolla at balloon stage. (d) Several petals separate. (e) Corolla at bell-shaped stage. (f) Full flowering. (g) First petals fading or falling. (h) Most petals dry or fallen.





**Supplementary Figure 4.** Pollination process. (a) Bee pollination. (b) Pollination experiment (1: controller box; 2: lifer; 3: pollinator; 4: electrostatic generator; 5: adjustable power supply; 6: computer). (c) Pollinator in work. (d) Kiwifruit flower after pollination.


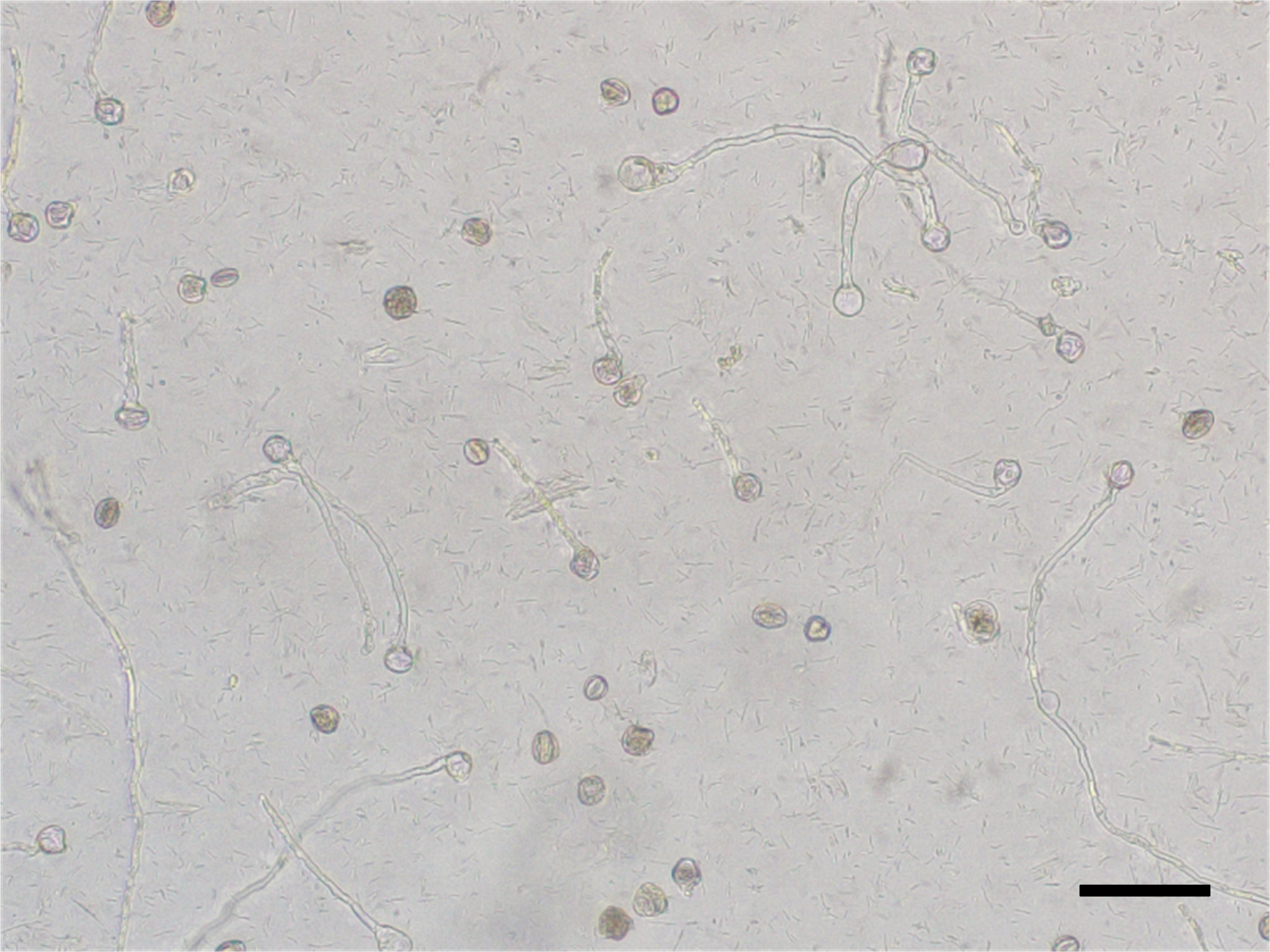


**Supplementary Figure 5.** Kiwifruit pollen in vitro germination without electrostatic treatment. Scale bar: 100 μm





**Supplementary Figure 6.** Fluorescence observation of pollen tubes after electrostatic pollination in styles of Xuxiang kiwifruit cultivars. (a) 14 kV; (b) 20 kV, the scale bar is 200 μm.
